# Amiodarone-Induced Epididymitis: A Case Report and Review of the Literature

**DOI:** 10.7759/cureus.62861

**Published:** 2024-06-21

**Authors:** Blake A Lyon, David Wynne

**Affiliations:** 1 Medicine, Edward Via College of Osteopathic Medicine, Auburn, USA; 2 Internal Medicine, Grandview Medical Center, Birmingham, USA; 3 Summit Internal Medicine, Grandview Medical Center, Birmingham, USA

**Keywords:** amiodarone-induced epididymitis, recurrent epididymitis, desethylamiodarone, epididymyalgia, scrotal pain, testicular pain, amiodarone, case report, epididymitis, side effects of amiodarone

## Abstract

Amiodarone is commonly used to prevent and treat life-threatening cardiac arrhythmias. However, it is also known to have an extensive side effect profile. A rare adverse effect of amiodarone is epididymitis. Epididymitis is inflammation of the epididymis that causes moderate pain in the posterior scrotum. The patient, in this case, developed left scrotal pain seven months after starting amiodarone and presented with symptoms consistent with epididymitis. The patient’s work-up included urinalysis with culture, treatment with antibiotics, and testicular ultrasound before being diagnosed with amiodarone-induced epididymitis. This diagnosis led to the discontinuation of amiodarone, which resulted in the complete resolution of the patient’s symptoms within two weeks. This case report is intended to increase awareness of epididymitis as a possible adverse effect of amiodarone and to stress the importance of considering this when there are no apparent anatomical or infectious causes of epididymitis.

## Introduction

Amiodarone is an iodinated benzofuran derivative classified as a class III antiarrhythmic. It is used to treat atrial and ventricular arrhythmias [1,2]. Amiodarone has an extensive adverse effect profile that can affect multiple organ systems. Common adverse effects of amiodarone include nausea, vomiting, and taste disturbances; moreover, amiodarone has also been documented to cause toxicities to the thyroid gland, lungs, liver, heart, eyes, and skin [2]. A rare adverse effect of amiodarone is epididymitis, occurring in less than 1% of patients taking the medication [3].

Epididymitis is an inflammation of the epididymis that can be a result of infectious or noninfectious causes, including sexually transmitted infections, urinary reflux due to bladder outlet obstruction, trauma, drug-induced, tuberculosis, and systemic inflammatory disease [4,5]. The etiology of epididymitis is highly dependent on age. In young males below the age of 14, epididymitis is thought to be related to anatomic abnormalities resulting in urinary reflux into the ejaculatory ducts or to be part of a postinfectious syndrome from *Mycoplasma pneumoniae*, enteroviruses, or adenoviruses [4]. In sexually active males between 14 and 35 years of age, *Chlamydia trachomatis* and *Neisseria gonorrhoeae* are the most common cause of epididymitis. In men older than 35 years of age, the most common cause of epididymitis is the retrograde flow of urine infected with bacteria such as *Escherichia coli* into the ejaculatory ducts by urinary reflux due to bladder outlet obstruction [4,5]. Noninfectious causes of epididymitis are thought to be the result of reflux of sterile urine into the vas deferens due to bladder outlet obstruction, underlying systemic disease, or an adverse reaction to a medication such as amiodarone [2-5].

The pathogenesis of amiodarone-induced epididymitis is poorly understood, and the diagnosis is made clinically after other possible causes have been excluded. Amiodarone and its metabolite (desethylamiodarone) can concentrate up to 3,000 times higher in testicular tissue than the plasma concentration; the reason for this high testicular concentration of amiodarone is unclear [6,7].

## Case presentation

A 78-year-old White male presented to the clinic with two weeks of left scrotal pain. He stated that the pain was constant, had progressively worsened since the onset, and was without radiation. He denied fever, chills, dysuria, flank pain, urethral discharge, and urinary retention. His past medical history included paroxysmal atrial fibrillation, coronary artery disease, chronic systolic heart failure, hypertension, and benign prostatic hyperplasia. His surgical history included coronary artery bypass grafting and automatic implantable cardioverter defibrillator/pacemaker placement. His medications included allopurinol, amiodarone 200 mg twice daily, aspirin, clopidogrel, enalapril, furosemide, metoprolol, spironolactone, tamsulosin, and warfarin. He had no known drug allergies at that time. He admitted to minimal alcohol use but denied tobacco or illicit drug use. He was married, recently retired, and denied any recent sexual activity. He reported a family history of cerebrovascular accident in his father and myocardial infarction in his mother but denied any other significant family history.

Vital signs on presentation were as follows: temperature of 97.4°F, blood pressure at 174/69 mmHg, heart rate of 61 beats/minute, 16 respirations/minute, oxygen saturation at 98% on room air, weight of 196 pounds, height of 5 feet 11 inches, and body mass index of 27.3 kg/m^2^. His physical examination was mostly unremarkable; however, his left scrotum was notably edematous and profoundly tender, particularly to the posterior aspect overlying the epididymis. The left testis was noted to be in a normal anatomic position. He was circumcised, and no genital lesions or penile discharge were noted. He had no costovertebral angle or suprapubic tenderness upon palpation.

The lab results, displayed in Table 1, were within normal limits except for the eosinophils, which were mildly elevated. The urinalysis results in Table 2 were unremarkable, and the urine culture revealed no bacterial growth. Since the patient reported his pain as progressively worsening and not acute in onset, along with normal positioning of his left testicle, testicular torsion seemed unlikely; however, testicular ultrasound was discussed to rule out testicular torsion definitively. The patient refused the ultrasound and was treated with a single intramuscular injection of 1 g of ceftriaxone as well as a 10-day prescription of oral doxycycline at 100 mg daily for potential bacterial epididymitis.

**Table 1:** Lab results, including CMP, CBC with differential, lipids, and thyroid studies All the lab results were within normal limits except for the eosinophils, which were mildly elevated CMP: comprehensive metabolic panel; BUN: blood urea nitrogen; eGFR: estimated glomerular filtration rate; IU: international unit; LDH: lactate dehydrogenase; AST: aspartate aminotransferase; SGOT: serum glutamic oxaloacetic transaminase; ALT: alanine aminotransferase; SGPT: serum glutamic-pyruvic transaminase; HDL: high-density lipoprotein; VLDL: very-low-density lipoprotein; LDL: low-density lipoprotein; TSH: thyroid stimulating hormone; MCV: mean corpuscular volume; MCH: mean corpuscular hemoglobin; MCHC: mean corpuscular hemoglobin concentration; RDW: red cell distribution width

Test	Result	Limits
CMP		
Glucose, serum	82 mg/dL	65-99 mg/dL
Uric acid, serum	6.2 mg/dL	3.7-8.6 mg/dL
BUN	10 mg/dL	6-20 mg/dL
Creatinine, serum	1.05 mg/dL	0.76-1.27 mg/dL
eGFR	90 mL/min/1.73	>59 mL/min/1.73
BUN/creatinine ratio	10	8-19
Sodium, serum	140 mmol/L	134-144 mmol/L
Potassium, serum	3.9 mmol/L	3.5-5.2 mmol/L
Chloride, serum	99 mmol/L	97-108 mmol/L
Carbon dioxide, total	24 mmol/L	18-29 mmol/L
Calcium, serum	9.6 mg/dL	8.7-10.2 mg/dL
Protein, total, serum	7.2 g/dL	6.0-8.5 g/dL
Albumin, serum	4.7 g/dL	3.5-5.5 g/dL
Bilirubin, total	0.3 mg/dL	0.0-1.2 mg/dL
Bilirubin, direct	0.11 mg/dL	0.00-0.40 mg/dL
Alkaline phosphatase	55 IU/L	39-117 IU/L
LDH	182 IU/L	0-225 IU/L
AST (SGOT)	24 IU/L	0-40 IU/L
ALT (SGPT)	22 IU/L	0-44 IU/L
Lipids		
Cholesterol, total	163 mg/dL	100-199 mg/dL
Triglycerides	55 mg/dL	0-149 mg/dL
HDL cholesterol	64 mg/dL	>39 mg/dL
VLDL cholesterol	11 mg/dL	5-40 mg/dL
LDL cholesterol	88 mg/dL	0-99 mg/dL
Thyroid studies		
TSH	0.888 uIU/mL	0.450-4.500 uIU/mL
T4, free (direct)	1.39 ng/dL	0.82-1.77 ng/dL
CBC with differential		
WBC	5.3 × 10^3^/uL	3.4-10.8 × 10^3^/uL
RBC	4.66 × 10^6^/uL	4.14-5.80 × 10^6^/uL
Hemoglobin	14.1 g/dL	12.6-17.7 g/dL
Hematocrit	41.5%	37.5-51.0%
MCV	89 fL	79-97 fL
MCH	30.3 pg	26.6-33.0 pg
MCHC	34.0 g/dL	31.5-35.7 g/dL
RDW	13.1%	12.3-15.4%
Platelets	163 × 10^3^/uL	150-379 × 10^3^/uL
Neutrophils	43%	40-74%
Lymphocytes	43%	14-46%
Monocytes	7%	4-12%
Eosinophils	6% H	0-5%
Basophils	1%	0-3%
Neutrophils (absolute)	2.3 × 10^3^/uL	1.4-7.0 × 10^3^/uL
Lymphocytes (absolute)	2.3 × 10^3^/uL	0.7-3.1 × 10^3^/uL
Monocytes (absolute)	0.3 × 10^3^/uL	0.1-0.9 × 10^3^/uL
Eosinophils (absolute)	0.3 × 10^3^/uL	0.0-0.4 × 10^3^/uL
Basophils (absolute)	0.0 × 10^3^/uL	0.0-0.2 × 10^3^/uL
Immature granulocytes	0%	0-2%
Immature granulocytes (absolute)	0.0 × 10^3^/uL	0.0-0.1 × 10^3^/uL

**Table 2:** Urinalysis results The urinalysis results were unremarkable

Test	Result	Limits
Urinalysis gross exam		
Specific gravity	1.016	1.005-1.030
pH	7.0	5.0-7.5
Urine color	Yellow	Yellow
Appearance	Clear	Clear
WBC esterase	Negative	Negative
Protein	Negative	Negative/trace
Glucose	Negative	Negative
Ketones	Negative	Negative
Occult blood	Negative	Negative
Bilirubin	Negative	Negative
Urobilinogen	1.0 mg/dL	0.0-1.9 mg/dL
Nitrite, urine	Negative	Negative
Microscopic examination		
WBC	0-5/hpf	0-5/hpf
RBC	0-2/hpf	0-2/hpf

**Figure 1:**
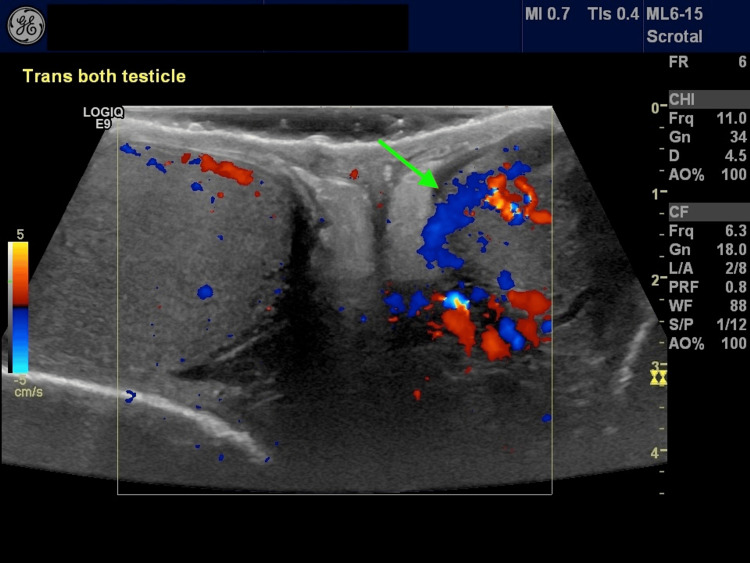
Testicular ultrasound showing marked hyperemic left epididymis consistent with epididymitis (green arrow)

He was also instructed to return to the clinic if his symptoms did not improve. Three days later, the patient called the clinic, stating that he had no improvement in his symptoms and reported a slight worsening of his pain. He was provided an additional 10-day prescription of trimethoprim/sulfamethoxazole at 160/800 mg every 12 hours, and after further discussion, he was agreeable to the testicular ultrasound. As shown in Figure 1, the testicular ultrasound revealed marked hyperemic left epididymis consistent with epididymitis and no evidence of testicular torsion or neoplasm. His lack of improvement and his worsening epididymitis despite three days of treatment suggested an alternative explanation for his epididymitis. He had been started on amiodarone seven months before his initial visit following percutaneous transluminal coronary angioplasty to his left anterior descending artery and subsequent atrial fibrillation. After a discussion with his cardiologist and other cardiologists (none of whom had ever seen amiodarone-induced epididymitis), the amiodarone was discontinued, and sotalol was started. His symptoms rapidly improved with near complete resolution after 14 days.

Nine years later, the patient presented to the clinic with recurrent left scrotal pain over a three-week period. The pain was the same as the previous instance, and he reported that his new cardiologist recently started him back on amiodarone. He said he had a follow-up appointment with his cardiologist in two days, so he was advised to stop taking the amiodarone and discuss different medication options with his cardiologist. Three weeks later, after his cardiologist changed his antiarrhythmic medication to digoxin, his symptoms had completely resolved.

## Discussion

Amiodarone-induced epididymitis presents with chronic epididymyalgia in the absence of fever or leukocytosis and has a variable presentation time that ranges from 4 to 71 months after starting amiodarone [1,7,8]. The scrotal pain may be unilateral or bilateral, with more severe pain on one side [6,8,9]. The natural history of amiodarone-induced epididymitis is highly variable but usually requires discontinuation [7,10] or amiodarone dose reduction [9,11]. In one case report, the patient suffered from amiodarone-induced epididymitis for 11 weeks, eventually requiring a bilateral epididymectomy. Following the epididymectomy, amiodarone-like crystals were identified in the epididymis, similar to those seen in skin changes associated with amiodarone [6]. Patients suspected to have amiodarone-induced epididymitis should be treated with dose reduction or cessation of amiodarone in favor of another antiarrhythmic medication. Consistent with our case, symptoms usually resolve within 10 days to three months following the discontinuation of amiodarone. The variability in resolution time is consistent with the slow elimination of amiodarone, which ranges from 25 to 110 days [1]. However, amiodarone-induced epididymitis may occur in patients where it is necessary to continue treatment with amiodarone. In a different case report, the cardiologist determined that it was necessary to continue amiodarone and treat the symptoms with analgesics. Following analgesic therapy, the symptoms resolved, but epididymitis was still present on radiologic imaging. This shows that treating patients symptomatically may be possible if discontinuing amiodarone is not an option [8,12]; however, this does not resolve the underlying issue.

Amiodarone-induced epididymitis is a diagnosis of exclusion. Our patient had a negative urinalysis and urine culture; bacterial infection seemed unlikely. Furthermore, our patient did not respond to ceftriaxone and doxycycline. The patient had no skin, joint, or other findings on physical examination that would suggest systemic disease. Tuberculosis can cause a chronic infection that would not have improved with the treatment he was given but seemed an unlikely culprit without risk factors for acquiring tuberculosis infection. The patient’s symptoms improved rapidly within two weeks following the discontinuation of amiodarone, reoccurred nine years later when amiodarone was restarted, and resolved within three weeks of discontinuing amiodarone the second time. These findings confirmed the diagnosis of amiodarone-induced epididymitis.

## Conclusions

In conclusion, this case highlights the significance of medications as the possible culprit of epididymitis. In the absence of anatomical or infectious suspicions, medications such as amiodarone should be considered. Amiodarone has a diverse side effect profile, but epididymitis is not as well known. Physicians should be aware of this rare adverse effect of amiodarone to prevent unnecessary treatment with antibiotics, and reduction or discontinuation of amiodarone should be considered. This recommendation should improve patient outcomes by aiding in quicker diagnosis and resolution of symptoms in patients suffering from amiodarone-induced epididymitis.

## References

[1] Colunga Biancatelli RML, Congedo V, Calvosa L, Ciacciarelli M, Polidoro A, Iuliano L (2019). Adverse reactions of amiodarone. J Geriatr Cardiol.

[2] Hamilton D Sr, Nandkeolyar S, Lan H (2020). Amiodarone: a comprehensive guide for clinicians. Am J Cardiovasc Drugs.

[3] Goldschlager N, Epstein AE, Naccarelli GV, Olshansky B, Singh B, Collard HR, Murphy E (2007). A practical guide for clinicians who treat patients with amiodarone: 2007. Heart Rhythm.

[4] Mcconaghy JR, Panchal B (2016). Epididymitis: an overview. Am Fam Physician.

[5] Taylor SN (2015). Epididymitis. Clin Infect Dis.

[6] Shen Y, Liu H, Cheng J, Bu P (2014). Amiodarone-induced epididymitis: a pathologically confirmed case report and review of the literature. Cardiology.

[7] Dasu N, Khalid Y, Panuganti S, Daly S (2019). Amiodarone induced epididymo-orchitis. Urol Case Rep.

[8] Cicek T, Cicek Demir C, Coban G, Coner A (2014). Amiodarone induced epididymitis: a case report. Iran Red Crescent Med J.

[9] Nikolaou M, Ikonomidis I, Lekakis I, Tsiodras S, Kremastinos D (2007). Amiodarone-induced epididymitis: a case report and review of the literature. Int J Cardiol.

[10] Ibsen HH, Frandsen F, Brandrup F, Møller M (1989). Epididymitis caused by treatment with amiodarone. Genitourin Med.

[11] Kirkali Z (1988). Amiodarone-induced sterile epididymitis. Urol Int.

[12] Gabal-Shehab LL, Monga M (1999). Recurrent bilateral amiodarone induced epididymitis. J Urol.

